# Bio-inks with PRF Increase Human Osteosarcoma Cell Line (SaOS-2) Viability in Extrusion-Based 3D-Bioprinted Constructs

**DOI:** 10.1007/s10439-026-04023-x

**Published:** 2026-02-25

**Authors:** Viviana Claudia Torres-Ambolumbet, Manuel Santiago Ocampo-Terreros, Lina María Anaya-Sampayo, Dabeiba-Adriana García-Robayo

**Affiliations:** 1 School of Engineering, Pontificia Universidad Javeriana, Bogotá, Colombia; 2 Dentistry Research Center, School of Dentistry, Pontificia Universidad Javeriana, Bogotá, Colombia; 3 Buccal Department, School of Dentistry, Pontificia Universidad Javeriana, Bogotá, Colombia; 4 Faculty of Dentistry, Centro de Investigación e Innovación Biomédica (CiiB), Universidad de Los Andes, Santiago, Chile; 5 Department of Civil Engineering, Pontificia Universidad Javeriana, Bogotá, Colombia

**Keywords:** 3D bioprinting, Bioinks, Viability, Platelet concentrates, Tissue regeneration

## Abstract

**Purpose:**

The growing demand for functional tissues and organs has driven advances in tissue engineering, particularly through 3D bioprinting. However, the mechanical stress associated with extrusion can compromise cell viability, limiting its clinical applicability. This study aimed to evaluate the viability of mature osteoblast-like cells (SaOS-2) in alginate-based bioinks supplemented with different platelet concentrates, platelet-rich plasma (PRP), platelet-poor plasma (PPP), platelet-rich fibrin (PRF), and injectable PRF (iPRF) to identify formulations that enhance cell survival post-printing.

**Methods:**

Bioinks composed of alginate and varying concentrations (10% and 20%) of platelet concentrates were prepared and characterized rheologically. SaOS-2 cells were embedded in the bioinks and printed using extrusion-based 3D bioprinting. Printed scaffolds were analyzed for cell viability using the LIVE/DEAD assay and confocal microscopy at 24, 48, and 72 hours post-printing.

**Results:**

Rheological analysis confirmed the printability of constructs containing 10% PPP, 10% PRF, and 20% PRF. Cell viability exceeded 58% at 24 hours and 80% at 48 hours across all tested bioinks. Notably, PRF-containing constructs demonstrated viability recovery up to 86% at 72 hours, suggesting a protective and regenerative role.

**Conclusion:**

PRF-enriched bioinks significantly improve cell viability after extrusion and enhance the physical integrity of bioprinted scaffolds. These results support the potential of PRF-based bioinks as promising candidates for clinically relevant bone tissue engineering applications.

**Supplementary Information:**

The online version contains supplementary material available at https://doi.org/10.1007/s10439-026-04023-x.

## Introduction

Bone regenerative capacity can be affected by chronic diseases such as osteoporosis or osteoarthritis, which are particularly prevalent in people over 50 years old, affecting approximately 19.7% of the population in developed countries [1]. Additionally, the size of the traumatic lesion (greater than 2.5 cm) also influences the regenerative process [2]. On the other hand, motor vehicle collisions contribute significantly to bone injuries, with an estimated 40 to 50 million people worldwide suffering non-lethal trauma each year [3]. Given these challenges, there is a pressing need to explore advanced strategies such as tissue engineering to overcome the limitations of natural bone regeneration.

Tissue engineering is a discipline that combines the principles of biology, engineering, and material science to repair and even regenerate injured tissues. To this end, it is based on a triad composed of *i*) biomaterial use to generate 3D structures mimicking the extracellular matrix, *ii)* cells in an undifferentiated state, such as stem cells, and *iii*) biologically active molecules that favor cell adhesion, migration, and even cell differentiation [4]. Scaffold fabrication, or the generation of 3D structures, can be performed through conventional methods, such as electrospinning and lyophilization. However, it is challenging to control characteristics, such as pore size and homogeneous cell distribution, which are indispensable for tissue regeneration [5].

To overcome these limitations, 3D bioprinting has emerged as a promising technique in regenerative medicine, enabling the spatial control of biomaterials and cells to fabricate constructs with defined architecture and uniform cell distribution [6, 7]. In extrusion-based bioprinting, natural polymers such as gelatin, hyaluronic acid, and sodium alginate are commonly used. Among these, sodium alginate stands out due to its biocompatibility, adjustable viscosity, ease of preparation, and rapid ionic crosslinking in the presence of calcium ions [8].

Nevertheless, a significant challenge of 3D bioprinting lies in preserving cell viability during and after extrusion. It requires the application of stress to the material, with factors such as shear stress, the viscosity of the bioink, intra-channel pressures, extrusion speed, and temperature affecting cell viability [9]. These physical factors can lead to cellular processes such as pyknosis, karyolysis, or apoptosis, presenting significant challenges to achieving successful regeneration outcomes [10].

To address this, diverse strategies have been developed to improve cell viability in bio-printed constructs, utilizing techniques such as cell embedded within the biomaterial, typically using natural polymers with high permeability to oxygen, nutrients, and water-soluble compounds, such as gelatin, collagen, and sodium alginate [11]. Additionally, studies have suggested that co-polymers, including poloxamer, employed in encapsulation techniques, have demonstrated cell viability of up to 70% after 24 hours of bioprinting and 85% two weeks after bioprinting [12]. However, despite these advances, the search for biologically active components that can further enhance cell viability in bioinks [13].

In this context, platelet concentrates (PCs) have emerged as a cost-effective, autologous source of growth factors and cytokines that support cell adhesion, proliferation, and differentiation. Their intrinsic biological properties, along with favorable pH and viscosity profiles, make them attractive candidates to protect cells during the extrusion process [14]. Platelet concentrates can be classified into three generations: Platelet-rich plasma (PRP) and Platelet-poor plasma (PPP) correspond to the first generation, while Platelet Rich Fibrin (PRF) corresponds to the second generation. Injectable platelet-rich fibrin (iPRF) corresponds to a third-generation product, whose use is becoming increasingly frequent in diverse medical applications [15].

Therefore, this study aimed to evaluate whether the incorporation of different PCs (PRP, PPP, PRF, and iPRF) into sodium alginate-based bioinks improves the viability of the human osteoblast-like cell line (SaOS-2) following extrusion-based 3D bioprinting. In this study, platelet concentrates were incorporated not as physical encapsulating agents but as biological supplements embedded within the hydrogel matrix, releasing growth factors and cytokines that avoid cellular damage from extrusion-induced shear stress. We hypothesized that platelet concentrates could mitigate extrusion-induced mechanical stress through biochemical mechanisms, principally the sustained release of growth factors, which promote cellular survival during and after printing.

## Materials and Methods

This study received approval from the Pontificia Universidad Javeriana School of Dentistry Ethics Research Committee Act 006, 2017. Venous samples from two healthy non-smoking donors (a 25-year-old woman and a 28-year-old man) who had not consumed medications in the last three months were collected after signing informed consent. 150,000 and 400,000 platelets/mm^3^ of blood. Peripheral blood (43 mL per donor per procedure) was collected from a single venipuncture using sterile vacutainer tubes without anticoagulant (n = 16 tubes that correspond to eight tubes per participant) or with sodium citrate (n = 12 tubes that correspond to six tubes per participant), depending on the platelet concentrate to be prepared. Each sample was processed immediately after collection and divided into multiple tubes to obtain PRP, PPP, PRF, and iPRF. The platelet concentrates obtained from these two donors were used fresh within one hour of preparation, without freezing or long-term storage.

### Platelet Concentrates (PCs)

For all platelet concentrates, two blood draws were performed biweekly for each participant: three tubes with anticoagulant (PRP and PPP) and four tubes without anticoagulant (PRF and iPRF) in each drawing.

#### Platelet-Rich Plasma (PRP) and PLATELET-Poor Plasma (PPP):

Blood sample was collected in sodium citrate tubes (Beckton Dickinson Vacutainer, Franklin Lakes, NJ, USA) and immediately centrifuged at 1,200 rpm for 8 minutes. Following this, the supernatant and buffy coat (comprising plasma and leukocytes) were collected and centrifuged again under the same parameters, where the supernatant was approximately 1 mL of PPP/per tube was used to prepare a pool of twelve tubes obtained from the two participants, and the buffy coat was PRP with similar quantities as PPP across all tubes [16].

#### Platelet-rich Fibrin (PRF)

A blood sample was collected in tubes without anticoagulant (Beckton Dickinson Vacutainer, Franklin Lakes, NJ, USA) and immediately centrifuged at 2,700 rpm for 12 minutes. To allow for fibrin network formation in platelet-rich fibrin, the sample was allowed to incubate at room temperature for 5 minutes. Each 7 mL tube yielded approximately 1.0–1.2 mL of PRF before dehydration. Due to its gel-like characteristic, the fibrin clot was first frozen at –70 ºC for 24 h and then placed in a lyophilizer (FreeZone 4.5 Benchtop Freeze Dry System) at –51 ºC for an additional 24 h to obtain the lyophilized PRF. After lyophilization, the PRF was stored until its use; each lyophilized tube was resuspended in 4 mL of culture medium, and then a pool of eight tubes without anticoagulant was prepared, four tubes per participant [17, 18]. This process did not affect PRF biological properties, as described by Li and collaborators, where lyophilized L–PRF was used as a supplement in periodontal ligament, dental follicle stem cell, and alveolar bone primary cell culture, with viabilities exceeding 100% [19].

#### Injectable Platelet-Rich Fibrin (iPRF)

A blood sample was collected in tubes without anticoagulant (Beckton Dickinson Vacutainer, Franklin Lakes, NJ, USA) and immediately centrifuged at 800 rpm for 4 minutes, and then two layers were observed: one corresponding to iPRF and a red blood cell layer, which was discarded [20]. 1 mL of iPRF/per tube, and then a pool of the remaining eight tubes was prepared.

### Cell Culture

Mature osteoblasts (osteosarcoma cell line SaOS-2 ATCC HTB-85) were cultured at 37 °C under 5% CO_2_ incubation conditions in high glucose DMEM (LONZA), supplemented with 10% fetal bovine serum (FBS, Gibco) and 1% penicillin-streptomycin-amphotericin solution (R&D Systems).

### Cell Viability in 2D Culture

Cells were seeded at 5 × 10^3^ in a 96-well plate and allowed to adhere for 24 h. Following this, the media was replaced with DMEM supplemented with the two participants’ pooled PRP, PPP, PRF, and iPRF at 10%, 20%, and 50% concentrations for 24 h. After this, MTS reagent (CellTiter 96 AQueous One Solution Cell Proliferation Assay, Promega) was added, following the manufacturer’s instructions. Reading was carried out at 490 nm. PCs that allowed cell viability greater than 100% were selected for the following phases. Values above 100% represent metabolic activity normalized to the 10% FBS control (set as 100%) and reflect increased mitochondrial activity rather than absolute cell number. Measurements were carried out in triplicate.

### Bio-ink Preparation and Rheological Evaluation

A 7% sodium alginate mix was prepared (alginic acid sodium salt from brown algae, CAS No. 9005-38-3) with each selected pool of PC (10% PPP and PRF, 20% PRP and iPRF at 10 and 20%). Control corresponded to 10% FBS. Bioink formulations containing 50% platelet concentrates (i.e., 50% v/v PRP, PPP, PRF, or iPRF) were excluded because this concentration produced low-viscosity, non-printable inks. A shear-rate viscosity curve was performed on the rheometer, and these 50% formulations showed viscosity values below the minimum required for stable filament extrusion (≤10 Pa·s at 1 s⁻^1^), confirming their inability to sustain continuous strands during printing. Bioink mixes were agitated for 24 h at 4 °C, and rheological measurements (shear rate sweep, rotational recovery, and frequency sweep) were performed using freshly prepared bioinks in a rotational rheometer (TA Instruments Rheometer Rheolyst AR2000EX, New Castle, USA), with a parallel plate geometry in a 25 mm flat plate. All measurements were registered with a 1.00 mm gap width between plates at 37 °C.

To determine the solid and liquid-like states of the different bio-inks, a dynamic sweep stress was performed at a shear strain range of 0.1 to 250 s^-1^ at a frequency of 1 Hz. Rotational recovery and frequency sweep were performed. To evaluate the bio-inks’ viscosity recovery, they were subjected to a shear rate  of 1 s^-1^ for 60 seconds, followed by a shear rate of 100 s^-1^ for 30 seconds. To evaluate the behavior of the bio-ink during printing, this process was performed three times at the maximum shear rate (100 s^-1^) and twice at the minimum shear rate. A frequency sweep was performed with a frequency range of 0.01 to 100 Hz at 37 °C and 1% strain. All rheological measurements were performed in duplicate (n = 2) using independent bioink preparations.

### 3D-Printed Constructs

Cell-free constructs were printed to evaluate the printability, filament stability, and structural fidelity of the different bioink formulations. These preliminary assays, performed without cells, allowed optimization of composition and identification of the formulations suitable for subsequent bioprinting with SaOS-2 cells. Before 3D printing was carried out, the bio-ink shape fidelity was assessed using the filament collapse test for the six selected bio-inks. To this end, the deflection of a suspended bio-ink filament on a platform with six pillars, spaced from 1 mm to 6 mm apart, was assessed. The filament fusion test was performed by extruding the material in a meandering pattern composed of parallel strands with increasing spacing, starting at 0.1 mm and increasing up to 2 mm. The ratio between the length of the fused filament (fs) and the thickness of the filament (ft) was calculated, with desirable fs/ft values ​​of 1 ± 0.5. However, 10% and 20% iPRF inks were excluded from the study, as their fs/ft values were 2.1 and 2.2, respectively, corresponding to low integrity and elasticity structures. The procedures for the filament collapse and filament fusion tests followed the methodology described by Ribeiro et al. and Schwab et al. Each formulation was evaluated in triplicate (n = 3), and the fs/ft ratios were expressed as mean ± standard deviation [21, 22]. Statistical analysis was performed using one-way ANOVA followed by Tukey’s post hoc test, with a significance level of p < 0.05. Based on rheological parameters obtained in the preceding section, the following bio-inks were selected: (i) Alg–20% PRP, (ii) Alg –10% PPP, (iii) Alg –10% PRF. As a control, Alg–10% FBS was used. Bio-printing was performed using a mechanical extrusion bioprinter (Regemat 3D, v1.0, Spain) in 10 mm × 10 mm × 3 mm constructs with 2 mm × 2 mm pores and a height of 0.4 mm. The only modifiable parameter was a 2 mm/s printing speed, with a 0.010-inch (0.254 mm) extrusion tip. Once printed, cross-linking of each construct was performed by exposing it to 2.5% calcium chloride (CAS No. 10043-52-4) at pH 7.3 for 5 minutes. Following, they were washed with PBS [23].

#### Swelling Assay

Swelling tests were performed by gravimetry in triplicate. Constructs were weighed to obtain an initial weight (W_0_), placed in a 12-well culture dish, and covered with DMEM. They were then incubated at 37 °C for 2, 4, 8, 10, and 24 hours. At each time point, constructs were washed with dH_2_O and weighed, obtaining W_f_ [24]. Equation 1 was used to calculate the swelling ratio.

1 $$Swelling : \frac{{W_{f} - W_{0} }}{{W_{0} }}*100\%$$

##### Degradation Rate Test

After construct lyophilization, each one was weighed to determine initial dry weight, W_0_, and placed into a well of a 12-well tissue culture dish with DMEM until they were completely covered. For each alginate PC, three wells were prepared. After 24 h of incubation, the media were removed, washed with PBS, and the final weight (W_f_) was recorded at 2, 4, 8, 10, 24, and 48 h [23]. Equation 2 was used to calculate the degradation rate.

2 $$Degradation : \frac{{W_{0} - W_{f} }}{{W_{0} }}*100\%$$

### Bio-Printing

After selecting the optimal formulations, SaOS-2 cells were incorporated into the bioinks and printed under standardized conditions established during the cell-free optimization stage. The final constructs were fabricated using the same scaffold geometry and printing parameters to ensure reproducibility for biological evaluation. For each selected bioink, a 7% alginate solution in DMEM culture medium containing 1 × 10^6^ SaOs-2 cells/mL with the pool of 20% PRP, 10% PPP, 10% PRF, or 10% FBS, respectively, was prepared at 37 °C for homogenization. Bioprinting, as mentioned earlier, was employed.

#### Scanning Electron Microscopy

After 24 hours of incubation, the bio-printed constructs were fixed with 2.5% glutaraldehyde and then lyophilized. They were then sputter-coated with gold particles for visualization using a Carl Zeiss Evo HD15 scanning electron microscope (SEM) at the Microscopy Laboratory of the Faculty of Engineering, Pontificia Universidad Javeriana. The microscope was operated at an acceleration voltage of 10 kV under high vacuum conditions (~10⁻^4^ Pa), and images were acquired at 55X and 850X magnifications.

##### Cell Viability in 3D Bio-Printed Constructs

Cell viability in bio-printed constructs was evaluated at 24, 48, and 72 hours using the Live/Dead (ab115347, Abcam) kit, following the manufacturer’s instructions. Briefly, after 24, 48, and 72 hours, the incubation constructs were washed with PBS, and EthD-1 and calcein AM were added. The constructs were then incubated for 2 hours at 37 °C. Cells were visualized using an Olympus IX71 optical fluorescence microscope with 485-495 nm excitation/emission for EthD-1 under 20X magnification. ImageJ was utilized to determine the live (green) and dead (red) cell numbers in three regions of the construct. The cell viability percentage was calculated using equation 3.

3 $$\% Viability = \frac{Live cells}{{Live cells + Dead cells}}*100$$

#### Statistical Analysis

Statistical analyses were performed using GraphPad Prism version 10 (GraphPad Software, San Diego, CA, USA). Descriptive statistics, including measures of central tendency and dispersion, were calculated. Comparisons between constructs were carried out using one-way ANOVA follows Tukey´s post hoc test, with statistical significance set at *p < *0.05. Data were reported as mean ± standard deviation.

## Results

### Cell Viability in 2D Culture

**Fig. 1:**
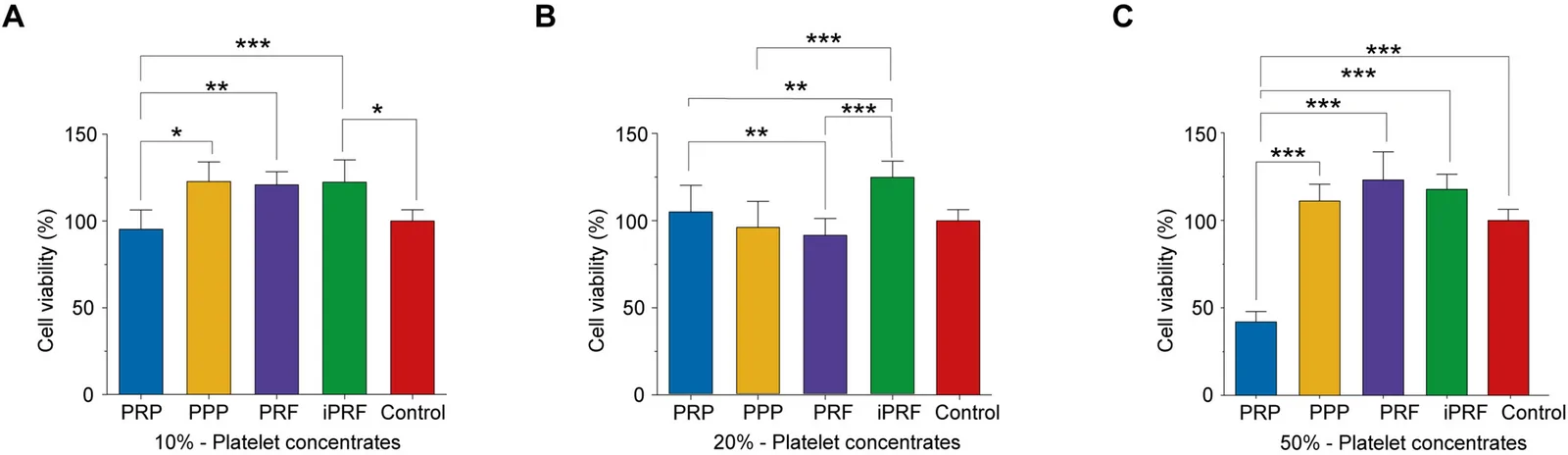
Effect of Platelet Concentrates on SaOS-2 Cell Viability in 2D Culture Cell viability was assessed using the MTS assay after 24 h of exposure to different concentrations of platelet concentrates (PCs) in DMEM. The control group consisted of DMEM supplemented with 10% fetal bovine serum (FBS), set as 100% viability. **A** 10% PCs: platelet-poor plasma (PPP), platelet-rich fibrin (PRF), and injectable platelet-rich fibrin (iPRF) showed viability > 100%. **B** 20% PCs: platelet-rich plasma (PRP) and iPRF reached 104% and 124% viability, respectively. **C** 50% PCs showed similar viability to 10% PCs. Statistical analysis was performed using one-way ANOVA followed by Tukey’s post hoc test for each PC group. *p < 0.05 **p < 0.01 ***p < 0.001. Data represent mean ± SD, n = 3. *PCs* platelet concentrates, *PRP* platelet-rich plasma, *PPP* platelet-poor plasma, *PRF* platelet-rich fibrin, *iPRF* injectable platelet-rich fibrin, *FBS* fetal bovine serum, *DMEM* Dulbecco’s Modified Eagle Medium. Values above 100% reflect metabolic activity relative to the FBS control and do not indicate an increase in absolute cell number

The effect of different concentrations of platelet concentrates (PCs) on the viability of SaOS-2 cells cultured in 2D was evaluated using the MTS assay after 24 h of exposure. As 100% viability was achieved when cells were cultured in DMEM supplemented with 10% fetal bovine serum (FBS). As illustrated in Figure 1A, 10% PPP, PRF, and iPRF achieved a cell viability greater than 100%. For 20% PRP and iPRF, viabilities were 104% and 124%, respectively. Last, similar results were obtained for 50% PCs when compared with 10%. To assess whether different concentrations within the same platelet concentrate produced distinct effects, one-way ANOVA with Tukey’s post hoc test was performed for each PC group. Significant differences were observed among PC types at the same concentration (Figure 1), including higher viability in iPRF, PPP, and PRF compared with PRP at 10% (p < 0.01) in addition, iPRF was higher compared to the others PC at 20% (p < 0.001), and lower viability for 50% PRP compared with the FBS control (p < 0.001). These values > 100% indicate higher metabolic activity relative to FBS control and should not be interpreted as an increase in absolute cell number.

### Rheological Characterization

**Fig. 2:**
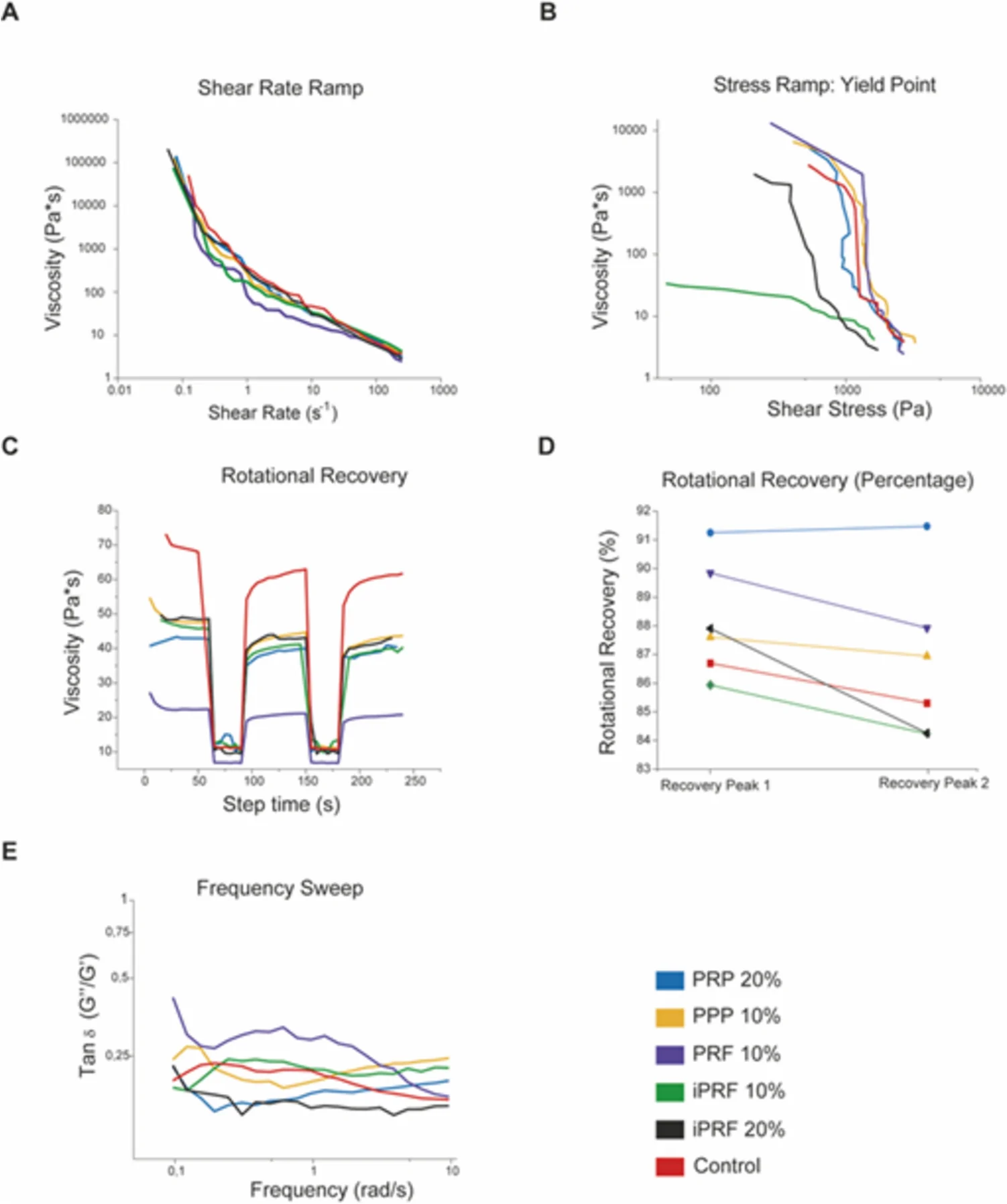
Rheological characterization of bioinks supplemented with platelet concentrates (PCs). **A** Shear rate sweep revealed pseudoplastic behavior in all selected bioinks, with 10% PRF and the FBS control showing higher viscosities. **B** Yield stress was determined via dynamic stress sweep, showing variability among PCs, with 10% PRF exhibiting the highest yield stress. **C–D** Rotational recovery tests showed all PC-supplemented bioinks recovered over 80% of their original viscosity post-extrusion, with 20% PRP displaying the highest recovery. **E** Frequency sweep confirmed solid-like behavior in all bioinks, with low Tan δ values indicating a dominant elastic response under dynamic deformation. Data represent mean ± SD, n = 2. *PCs* platelet concentrates, *PRP* platelet-rich plasma, *PPP* platelet-poor plasma, *PRF* platelet-rich fibrin, *iPRF* injectable platelet-rich fibrin, *FBS* fetal bovine serum, *Tan δ* tangent delta (G″/G′), *G′* storage modulus, *G″* loss modulus.

Bio-inks containing PCs at 50% were excluded from the study because rheological measurements showed that these formulations exhibited viscosity values below the measurable range for stable extrusion and did not reach a detectable yield stress during the shear-rate sweep. All selected bio-inks, including 10% and 20% PPP and PRF, as well as a 10% FBS control, were evaluated using a shear rate sweep, which revealed pseudoplastic behavior. Among them, 10% PRF and the FBS control exhibited higher viscosity values across low shear rates, indicating that supplementation with PCs did not alter shear-thinning behavior but contributed to increased viscosity in specific formulations (Figure 2A).

The yield stress was determined through a dynamic sweep stress test to assess the stress required to allow the ink to flow [25]. It was evidenced that 10% PPP presented yield stresses of 736 ± 65 Pa, 20% PRP of 858 ± 78 Pa, and 10% PRF of 1,326 ± 105 Pa. 10% and 20% iPRF presented yield stresses of 389 ± 49 Pa, whereas the control presented a yield stress of 922 ± 54 Pa. These results indicate that formulations with higher fibrin content (such as PRF) exhibit increased structural resistance before flow, whereas liquid-rich formulations (iPRF) show lower yield stresses (Figure 2B).

Furthermore, the rotational recovery refers to the bio-inks’ ability to recover their viscosity once the extrusion stress has ceased [25]. All PC-supplemented bioinks recovered more than 80% of their initial viscosity, with recovery values of 96% ± 4% for 20% PRP, 92% ± 5% for 10% PRF, 89% ± 6% for 10% PPP, 85% ± 5% for 20% iPRF, and 82% ± 7% for 10% iPRF. These results confirm that PC incorporation enhances thixotropic recovery, a key parameter for stabilizing printed filaments immediately after deposition (Figures 2C and 2D).

To evaluate the material behavior (liquid-like or solid-like) under oscillatory deformation, a frequency sweep test was performed [25]. The predominance of the storage modulus (G′) over the loss modulus (G″) indicates solid-like behavior, which is reflected in low Tan δ values (G″/G′), typically approaching 0. The following Tan δ values were obtained: 20% i-PRF (0.164 ± 0.012), 20% PRP (0.179 ± 0.024), control (0.202 ± 0.025), 10% PPP (0.220 ± 0.254), 10% PRF (0.218 ± 0.075), and 10% i-PRF (0.218 ± 0.049). All values remained below 0.25, indicating a dominant elastic (solid-like) response in all bioinks tested. These results confirm that the bio-printed constructs exhibit ideal rigidity under dynamic deformation conditions (Figure 2E).

### Bio-inks Printability

Bioink printability characteristics were evaluated through a filament collapse test to determine the maximum distance at which the filament remained stable and did not collapse. Additionally, the fusion test allowed for the assessment of the minimum distance at which two filaments did not fuse, thus selecting the ideal pore size [23, 26]. For the filament collapse tests, PRP-, PPP-, and PRF-based bioinks maintained filament integrity at spans up to 4 mm, whereas iPRF bioinks showed early collapse beginning at 1.5 mm. Because the collapse test produces qualitative/semi-quantitative outcomes (maximum stable unsupported distance), no inferential statistical analysis was applied, consistent with published protocols. These results are shown in Supplementary Figure 1. For the filament fusion test, the fs/ft ratio (mean ± SD) was calculated for each condition. Bioinks containing 10% PPP demonstrated an fs/ft ratio of 1.166 ± 0.257, followed by 10% PRF (1.534 ± 0.563) and 20% PRP (1.540 ± 0.326). In contrast, 10% and 20% iPRF formulations exhibited fs/ft ratios above 2.0 (2.171 ± 0.895 and 2.235 ± 0.987, respectively), indicating excessive filament spreading and poor structural fidelity. Statistical analysis for the fs/ft ratios was performed using one-way ANOVA with Tukey’s post hoc test (p < 0.05). These results are displayed in Supplementary Figure 1. Considering both assays, a 2 mm × 2 mm pore size was selected for subsequent experiments, as PRP, PPP, PRF, and the FBS control retained adequate fidelity at this spacing. In contrast, 10% and 20% iPRF bioinks were excluded from further bioprinting because their printability metrics fell outside acceptable ranges, showing early collapse at 1.5 mm (vs. 4 mm in other groups) and fs/ft values > 2.0 (vs. 1.1–1.5 for printable inks).

### Swelling and Degradation Rate Assays

**Fig. 3:**
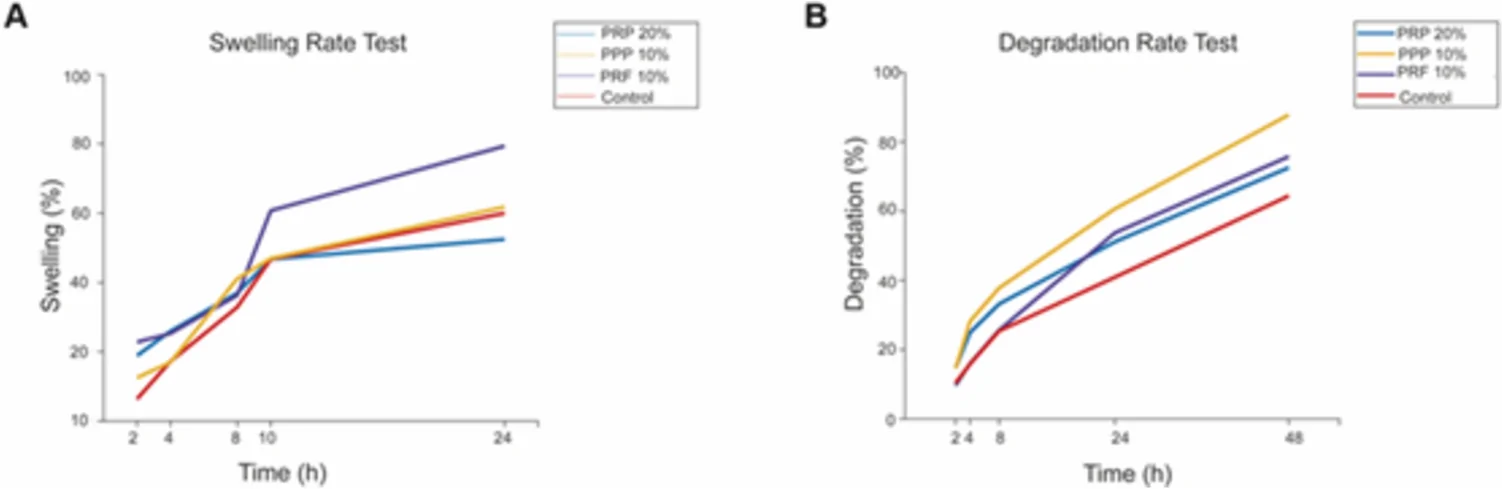
Swelling and degradation evaluation of 3D-printed constructs supplemented with platelet concentrates (PCs). **A** Swelling capacity was highest in 10% PRF constructs (~70%), followed by 10% PPP and FBS control constructs (~60%), and lowest in 20% PRP constructs (~52%). **B** Degradation rates after 48 h were greatest in 10% PPP constructs (~80%), while the other formulations exhibited lower mass loss values (70%). Data represent mean ± SD, n = 3. *PCs* platelet concentrates, *PRP* platelet-rich plasma, *PPP* platelet-poor plasma *PRF* platelet-rich fibrin, *FBS* fetal bovine serum

The swelling assay is fundamental because it helps establish its impact on nutrient and oxygen exchange with the extracellular matrix (ECM). Furthermore, the degradation rate test determines the construct’s decomposing time while new tissue is being formed [27]. The 10% PRF constructs were the most hydrophilic, reaching 70%, followed by the 10% PPP and control constructs at 60%. The lowest swelling was observed for the 20% PRP constructs with a swelling of 52% (Figure 3A). On the other hand, constructs with 10% PPP exhibited the highest degradation rate after 48 h of evaluation, with an 80% rate. The other constructs reached degradation rates lower than 70% (Figure 3B).

### Scanning Electron Microscopy

**Fig. 4:**
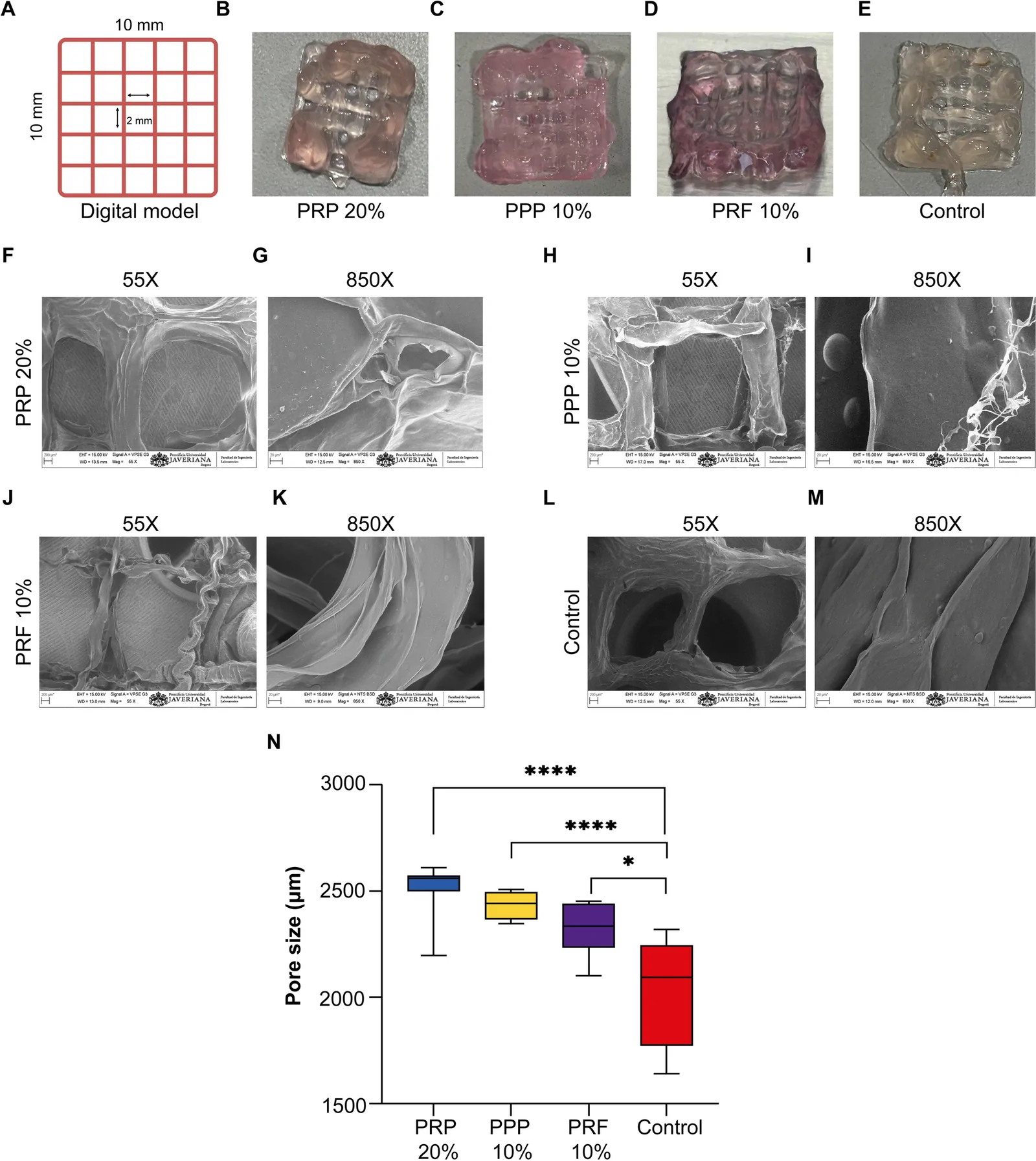
Morphological characterization of 3D-printed alginate constructs supplemented with platelet concentrates (PCs). **A** Digital model of the 10 × 10 × 3 mm construct with a 2 × 2 mm pore architecture. **B–E** Representative macroscopic images of printed scaffolds containing 20% PRP, 10% PPP, 10% PRF, and 10% FBS control, illustrating differences in filament definition, opacity, and surface density among bioinks. **F–M** SEM micrographs of 7% alginate constructs supplemented with 20% PRP **F–G**, 10% PPP **H–I**, 10% PRF **J–K**, and 10% FBS control **L–M.** Images were acquired at 55X **F, H, J, L** and 850X **G, I, K, M. N** Quantitative pore-size analysis (median ± SD) showed significantly larger pore diameters in PRP (2,512 ± 132.8 µm) and PPP (2,435 ± 62.32 µm) compared with the control (2,028 ± 245.3 µm), with PRF exhibiting intermediate values (2,319 ± 121.7 µm). Statistical differences: ****p < 0.0001 vs. control (PRP, PPP); *p < 0.01 vs. control (PRF). Representative images correspond to n = 3 constructs per group. *PCs* platelet concentrates, *PRP* platelet-rich plasma, *PPP* platelet-poor plasma, *PRF* platelet-rich fibrin, *FBS* fetal bovine serum, *SEM* scanning electron microscopy

Scanning electron microscopy was used to visualize bio-printed 7% alginate constructs with different platelet concentrates (PCs) (20% PRP, 10% PPP, and 10% PRF), as well as the control 10% FBS (Figure 4). To contextualize the SEM analysis, a digital model of the intended construct geometry was included (Figure 4A), along with representative macroscopic images of the printed scaffolds for each bioink formulation (Figure 4B–E), showing clear differences in surface appearance, opacity, and filament definition among PRP, PPP, PRF, and control bioinks. Through the SEM micrographs, it can be observed that the 10% PPP The 10% PPP, 20% PRP, and control groups presented similar morphologies, characterized by rough surfaces and granular structures, which likely correspond to platelets. For the 10% PPP, it was possible to observe a cell embedded within the material with a spherical form. On the other hand, the 10% PRF demonstrated the most diverse morphology, characterized by wave filaments, which is likely due to the lyophilization process (Figure 4 J). Granular forms were observed with a size less than 20 μm, probably corresponding to platelets (Figure 4 K). High-resolution SEM images at 55X and 850X magnification for all formulations (Figure 4F–M) were incorporated to illustrate differences in fibrillar alignment, pore wall structure, and surface texture among bioinks.

Quantitative pore-size analysis further confirmed morphological differences across groups. Constructs supplemented with 20% PRP showed the largest median pore diameter (2512 ± 132.8 µm), followed by 10% PPP (2435 ± 62.32 µm) and 10% PRF (2319 ± 121.7 µm), whereas the 10% FBS control exhibited the smallest pores (2028 ± 245.3 µm). Statistical comparisons indicated that PRP and PPP constructs had significantly larger pores than the control (****p < 0.0001 for PRP and PPP), and PRF also differed significantly (*p < 0.01). These findings show that platelet concentrates modulate scaffold microarchitecture by promoting greater pore expansion relative to the control bioink, with PRP producing the most pronounced effect (Figure 4N).

### SaOS-2 Construct Viability

**Fig. 5:**
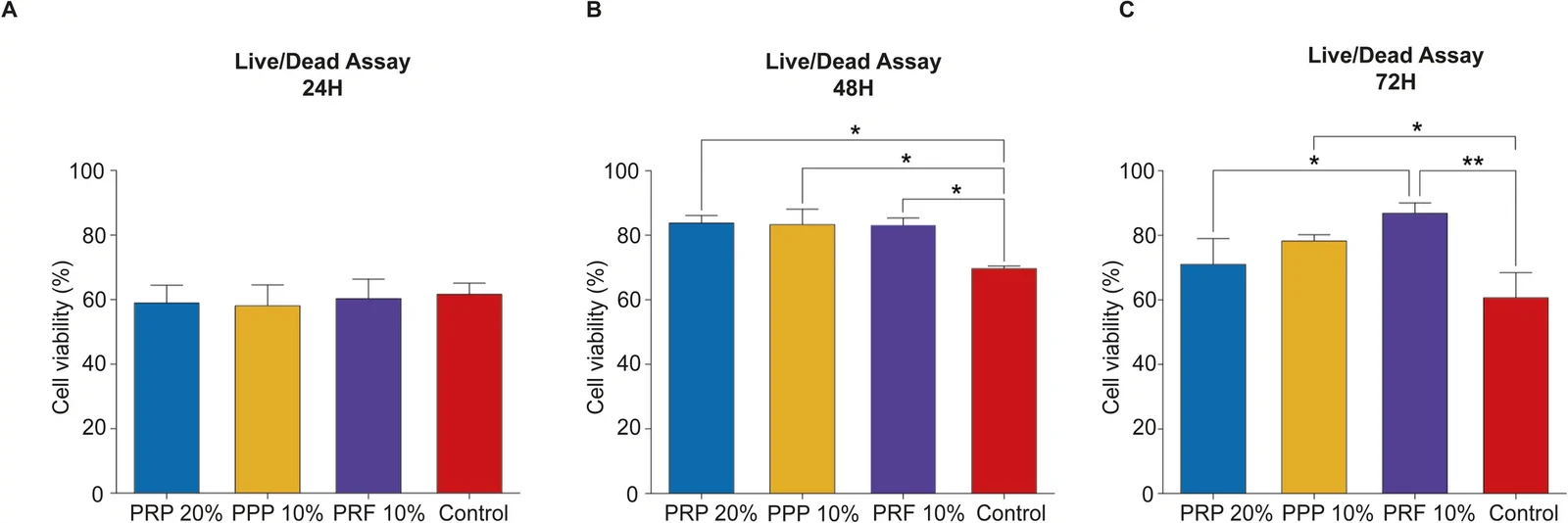
SaOS-2 cell viability in 3D bio-printed constructs supplemented with platelet concentrates (PCs). Cell viability was assessed at 24, 48, and 72 hours post-bioprinting using a viability assay.** A** At 24 h, constructs with 20% PRP, 10% PPP, and 10% PRF showed viability between 58% and 61%, with no significant differences.** B** At 48 h, all PC-supplemented constructs exhibited viabilities greater than 80%, significantly higher than the 70% viability observed in the 10% FBS control. **C** At 72 h, viability further increased, with 20% PRP at 70%, 10% PPP at 75%, and 10% PRF at 85%, the latter showing statistically significant improvement over the control. Statistical analysis was performed by one-way ANOVA (*p < 0.05, **p < 0.01). Representative images are shown in Supplementary Figure 2. Data represent n = 2 independent constructs per group. *PCs* platelet concentrates, *PRP* platelet-rich plasma, *PPP* platelet-poor plasma, *PRF* platelet-rich fibrin, *FBS* fetal bovine serum

SaOS-2 cell viability was evaluated at 24, 48, and 72 h in bio-printed constructs. Representative images were selected for each bio-ink (Supplementary Figure 2). At 24 hours following their bio-printing, constructs elaborated with 20% PRP, 10% PPP, and 10% PRF presented viabilities between 58% and 61%, with no significant difference. At 48 h, constructs with PCs presented cell viability greater than 80%, with significant differences compared to the control (70% viability). Lastly, at 72 hours, a gradual increase in viability was observed, with 20% PRP presenting a value of 70%, 10% PPP a value of 75%, and 10% PRF a value of 85%. The latter presented highly significant differences compared to the control, suggesting SaOS-2 recovered 72 h after the printing process (Figure 5).

## Discussion

In tissue regeneration, scaffolds must be three-dimensional structures that mimic the extracellular matrix (ECM), providing an adequate environment for cell adhesion and growth. They can be constructed using biocompatible and biodegradable materials that contain pores to facilitate nutrient exchange [6]. Different techniques have been developed, with 3D bio-printing being the most promising, as it mimics native tissue with high fidelity. Additionally, cells are simultaneously bio-printed with the biomaterial, ensuring uniform distribution. However, during the extrusion process, cells are subjected to shear stress that can damage their membranes, affecting their viability [28, 29]. Shear stress is directly influenced by the pressure exerted during the process, the nozzle diameter, and ink viscosity, particularly when the needle diameter is reduced to enhance print resolution [30]. Viscosity is a measure of a fluid’s resistance to flow. During extrusion bioprinting, high viscosity increases shear stress on the material. This elevated shear stress can potentially damage or rupture cell membranes, negatively impacting cell viability [31].

Furthermore, during bio-printing, highly viscous solutions require high pressure, which affects cell integrity [13]. Moreover, external non-controllable factors, such as temperature, can affect the cells. As a case in point, the *Regemat 3D* v1.0 bio-printer lacks a heating system to maintain cells at 37 °C. A study conducted by Ouyang and collaborators assessed the viability of murine stem cells (ES-R1) after a 3D bioprinting process using three gelatin concentrations (5%, 7.5%, and 10%) and 1% alginate. Their findings revealed 40% cell viability at the highest gelatin concentration and temperatures lower than 30 °C [32].

Although the extrusion process subjects cells to mechanical stresses, direct quantification of shear stress was not performed in this study due to the lack of an inline pressure sensor. However, an estimated range of shear stress values (100–250 Pa) was calculated based on the nozzle diameter (0.254 mm), flow rate (2 mm/s), and viscosity of the bioinks, using the Hagen–Poiseuille approximation, in accordance with previously reported values for alginate-based inks [33–35]. These levels are below the critical thresholds (>500 Pa) known to cause irreversible membrane damage or cell lysis in osteogenic cells [36]. Therefore, the observed post-printing viability above 80% across all groups supports that the printing parameters used in this study maintained shear stresses within a tolerable range for SaOS-2 cells.

Hence, different strategies have been developed to improve cell viability in bio-printed constructs through cell encapsulation, utilizing natural polymers with high oxygen permeability, nutrients, and water-soluble compounds. Among the most widely used are gelatin, collagen, and sodium alginate [11, 37]. Temirel et al. analyzed 4% alginate combined with 1%, 2%, or 3% gelatin bioinks, printed with 3T3 murine fibroblasts. They observed that only alginate bio-ink presented 70% viability after 24 hours of printing, while a combination with gelatin (encapsulated cells) attained 85% viability without losing material integrity. Therefore, material combination is emerging as a good alternative to maintain cell viability [38]. Likewise, Greco and collaborators evaluated the viability of human dermal fibroblasts (HDFs) after 48 hours of printing in a Reg4Life (REGEMAT) bio-printer. Their constructs integrated polyethylene glycol (PEGDA) and sodium alginate (SA), obtaining 82.64% viability [39]. However, the process of encapsulation does not guarantee cell contact with the matrix [40]. On the other hand, studies have suggested that co-polymers, such as poloxamer, are promising for cell encapsulation techniques, which have demonstrated up to 70% cell viability after 24 hours of bio-printing and 85% after two weeks of printing [12].

In the process of bio-printing platelet concentrates containing autologous growth factors, cell adhesion and even survival can be improved. Zhao et al. elaborated on a gelatin-alginate bio-ink in combination with PRP, demonstrating that it increased cell viability up to 90% for dermal fibroblasts and epidermal stem cells in primary culture after 1, 4, and 7 days of culture. In addition, it also favored migration and even angiogenesis. Likewise, Anaya and collaborators reported similar results, where lyophilized PRF was added to constructs of nanohydroxyapatite, chitosan, gelatin, and alginate, resulting in greater cell viability at 1, 3, and 7 days for both human dental pulp stem cells and differentiated osteoblasts [41]. Therefore, PCs are a potential alternative for the development of new bio-inks with greater cell viability and cell protection [42].

Our study differs from earlier reports that analyzed isolated platelet concentrates or focused solely on either rheological or biological aspects. Here, four platelet concentrates (PRP, PPP, PRF, and iPRF) were systematically compared within identical alginate-based bioink formulations and printing parameters. This comprehensive approach revealed that low PRF supplementation (10 %) simultaneously preserved printability and achieved the highest post-printing viability (≈ 85 %), while higher liquid-phase contents (50 % PCs) impaired structural fidelity. These results extend previous findings by integrating quantitative rheological characterization with cell viability outcomes, demonstrating, for the first time, a direct relationship between yield-stress recovery and cellular survival after extrusion. In our system, platelet concentrates were incorporated not as physical encapsulating agents, but as biological supplements embedded within the hydrogel matrix. Their role is to provide a biochemical microenvironment enriched with growth factors and cytokines that sustain cell viability during and after printing, helping cells recover from extrusion-induced mechanical stress rather than physically shielding them from it. Consequently, this work provides both mechanistic and methodological advances for developing platelet-enriched bioinks aimed at bone tissue applications. Taken together, our findings indicate that the 10% PRF formulation provides the best overall balance between rheological behavior, printability, and post-printing cell viability, and therefore represents the most promising candidate for further bone tissue engineering studies.

In the present study, an increase in cell viability over time was also observed. After 24 h of bio-printing, cell viability was between 58 and 60%, which increased at 48 h., where viability ranged between 68.29 and 88.09% with all PCs, as well as FBS, specifically 20% PRP and 10% PPP, which demonstrated the highest cell viability at 48 h (83.736% ± 2.201; 83.286% ± 4.615). However, it decreased at 72 h (70.961% ± 7.059, 74.897% ± 5.335). In contrast, PRF constructs demonstrated a gradual increase in cell viability at 48 h (83.068% ± 3.807) and 72 h (86.824% ± 2.919). This could be due to PRF releasing growth factors in a more controlled manner compared to PRP and PPP. However, this was a limitation in the study, as it was not quantified. Collectively, in PCs constructs cell viability was increased in comparison with 10% FBS control, with SaOS-2 cell line with an estimated 48 h population doubling time.

Similar findings regarding PRP and PPP have been reported by Del Amo and collaborators investigating plasma-based bioinks for skin dressing regeneration. They used 8% sodium alginate in combination with PRP and PPP in printed scaffolds with adult human dermal fibroblasts. Initially, PPP constructs had a viability of less than 75%, and PRP had a viability of less than 50%; however, on day four after bioprinting, the constructs exceeded 100% viability [43]. Daikuara et al. characterized *in vitro* a 3D bio-ink based on platelet lysate (PL) for its potential application in skin tissue engineering. They elaborated their inks with 15% methacrylated gelatin, 50% PL, and human dermal fibroblasts. Their cell viability was greater than 96% after 24 hours, with a continuous increase up to seven days [44].

Likewise, Danastri et al. presented similar findings on the proliferation of gingival fibroblasts with iPRF and PRP, evaluated by the MTT assay, without the use of DMEM. Greater viability was observed on days 1, 3, and 5 in cells treated with iPRF followed by PRP, suggesting the results were due to a greater amount of growth factors suspended in iPRF’s dense fibrin network [45]. On the other hand, Talebi et al. investigated PRP and PPP as potential substitutes for FBS in the culture of cancer cells, specifically T-cell-like cells (CCRF-CEM). Their study revealed that 2% and 5% PRP and PPP resembled the FBS control, while at 24 h, 15% PRP increased cell viability by greater than 250%, and 15% PPP exceeded 200% viability [46]. In contrast, in the present study, 50% PRP was found to be toxic to cells, decreasing cell viability by up to 42%. Similar findings were obtained by Tavassoli-Hojjati et al., who evaluated the viability of periodontal ligament fibroblasts using the MTT assay at 0.1, 5, and 50% PRP concentrations. At 24 h, 50% concentration was cytotoxic, with 25.84% viability and an average of 35.73% after seven days of evaluation [47]. Regarding PRF, Tsai and collaborators evaluated cell viability using the MTT assay on four cell lines: periodontal ligament stem cells (PDL), gingival fibroblasts (GF), a primary culture of oral epithelium (GNM), and tibia cell sarcoma (U2OS-HTB 96). When the media was supplemented with PRF at an unspecified concentration, viabilities were greater than 100% for GF (120%), PDL (130%), and U2OS (135%) compared to control media without supplementation. In conclusion, their U2OS result was like the one observed in our study with SaOS-2 cells [48].

Yi et al. studied PRF in bio-printed constructs composed of 2% alginate, 4% gelatin, and 50% iPRF aimed at promoting oral soft tissue regeneration. They demonstrated that these constructs exhibited complete pseudoplastic behavior, with viscosity values below 10^4^ Pa·s across a range of shear rates starting from 0 1/s [49]. Daikuara et al. also found similar results when analyzing 15% methacrylated gelatin with 50% PL, exhibiting the same pseudoplastic behavior with a viscosity of less than 10^4^ as a function of shear rate (1/s) of 0 [44]. Those results suggest that adding PCs equal to or greater than 50% decreases bioink viscosities as a function of shear rate, while those at 20% don’t exhibit these characteristics. Platelet concentrate not only affects cell behavior but also the properties of the bio-inks, which require specific rheological and printability characteristics [50–52]. Therefore, we performed dynamic sweep stress, thixotropic studies (rotational recovery) and frequency sweep. From the rheological analysis, it was evident that all bioinks, including those with 20% PRP, 10% PPP, 10% PRF, and 10% and 20% iPRF, as well as 10% control FBS, exhibited adequate bio-printing properties. For the sweep stress, when viscosity is high as a function of shear rate close to 0, it indicates an initial resistance to flux, which prevents deformations and collapse of the printed structure before secondary cross-linking [26]. All bio-inks exhibited a pseudoplastic behavior, highlighting 10 and 20% iPRF, 20% PRP, and 10% PPP, which presented greater viscosity, suggesting that PC supplementation did not alter the bio-ink’s pseudoplastic behavior.

A shear stress sweep was performed to determine the yield stress, defined as the minimum stress required for the ink to flow [25]. In this study, bio-inks containing first- and second-generation platelet concentrates, 20% PRP, 10% PPP, 10% PRF, as well as the 10% FBS control, exhibited yield stresses of approximately 736 Pa. In contrast, bio-inks supplemented with third-generation platelet concentrates, 10% and 20% iPRF, showed a lower elastic limit of 389 Pa. These results suggest that supplementation with liquid platelet concentrates can reduce the bio-inks’ yield stress. Similar findings were described by Mouser et al., who studied yield stress as a determinant factor in gelatin-methacryloyl (GelMA) hydrogel bioprinting for cartilage bio-printing. In their study, they found that with increasing GelMA concentration at 3 and 10%, the yield stress increased from 1.38 Pa to 48.2 Pa [53]. However, Gospodinova et al. In their research regarding bio-printing by extrussion of hydroxyethylcellulose (HEC) and alginate for cervical tumor model, indicated the yield stress inversely correlated with the relative alginate content, observing a lower yield stress (105 Pa) at 5% alginate, whereas a higher yield stress (145 Pa) was observed for 1% alginate concentrations [54].

Rotational recovery of a bioink refers to the material’s capacity to regain its viscosity once the stress is removed, as in the case of extrusion [25]. Supplementation with any PC can increase rotational recovery, as was evidenced in the present study, where all bio-inks with PCs recovered more than 80% of their viscosity. This is in agreement with Ahlfeld and collaborators, who described bio-inks composed of alginate and methylcellulose, when combined with PRP, as managing to regain complete viscosity [55]. On the contrary, bioinks without PCs demonstrated less viscous recovery, as evidenced in the study performed by Sanei et al., who synthesized and characterized an oxidized alginate-gelatin-silk fibroin bioink. It was observed that seven combinations of these components obtained an average recovery of their initial viscosity of 52% [51].

A frequency sweep is performed to determine whether a bioink behaves as a viscoelastic liquid or a solid-like gel [25]. Understanding solid-like gel behavior as the predominance of the shear storage (G’) over the loss moduli (G’’), reflected in Tan δ (G’’/G’) close to 0. In the present study, all bio-inks exhibited a solid-gel-like behavior, with a predominance of the storage moduli, which postulates them as optimal bio-inks for maintaining the shape of a digital model. Similar results were observed by Somasekharan et al., where bioinks fabricated with alginate dialdehyde–gelatin, with and without PRP, exhibited solid behavior with a predominance of the storage module. This suggests that the addition of PCs can cause greater solid behavior, as it presented a lower Tan δ than the bioink without PRP [56]. This observation can be inferred from the results obtained herein, with 20% PRP and 10% PPP bioinks presenting greater solid-like behavior due to the low Tan δ levels achieved. In contrast, the study by Temirel et al. demonstrated that 4% alginate bioinks, alone and in combination with 1% and 2% gelatin, exhibited a liquid-like behavior with a predominance of the loss modulus, resulting in a low fidelity of the bioprinted shape. In contrast, 3% gelatin bioinks exhibited a solid-like behavior, characterized by a predominance of shear storage, which is ideal for digital model bioink printing [38].

Bioink’s rheological characteristics are critical in determining its printability, which is considered the capacity to produce scaffolds with precise shape fidelity during the printing process [57]. The bio-inks’ evaluated rheological parameters were in agreement with expectations, exhibiting pseudoplastic behavior, rapid viscosity recovery after stress applications, and a solid feature that contributed to improving fidelity with the digital model. These rheological features were consistent with the printability data, where the selected bioinks (20% PRP, 10% PPP, 10% PRF, and the FBS control) generated 3D-printed constructs with well-defined 2 × 2 mm pores and preserved filament geometry, as confirmed by macroscopic images, SEM micrographs, and quantitative pore-size analysis (Figure 4).

It is also noteworthy that external factors, such as external extrusion pressure or temperature, can result in a better resolution of the printed filament. An illustration is the study performed by Ahmadi et al., who evaluated the printability of a 2% alginate and 5% gelatin bioink printed at 23 ºC, 24 ºC, and 25 ºC. It was observed that lower temperatures yielded low uniformity, which tended to fuse adjacent filaments, establishing 25 ºC as the optimal temperature [58].

Different bioink compositions can attain a better pore size resolution at shorter distances, as evidenced by Ribeiro and collaborators. They evaluated the behavior of poloxamer 407 and PEG bioink. In their study, they decreased the poloxamer concentration and increased the PEG concentration, obtaining pore sizes of up to 0.4 mm [23]. In addition, the supplementation of laponite (synthetic silicate nanoclay) to alginate dialdehyde and gelatin bioinks can help achieve smaller pore sizes, as evidenced by Cai and collaborators. They increased the laponite concentration from 0.5% to 1% and obtained better resolution with a 1 mm distance [59].

Swelling and degradation characteristics are essential in bio-printed constructs, as they are key parameters. For optimal nutrient diffusion, swelling should not be excessive, thus not compromising the scaffold’s integrity. Additionally, controlled degradation is crucial for facilitating tissue regeneration by ensuring the gradual degradation of the scaffold while new tissue is formed [60].

The swelling capacity of the PRF construct reached 70%, being the most hydrophilic. This result agrees with Mirhaj et al., who compared the physical, mechanical, and biological effects of PRF and A-PRF in polyacrylamide nanofiber dressings. They demonstrated that the addition of PRF or A-PRF increased the nanofibers’ hydrophilicity by 260% and 253%, respectively, compared to the control at 230%. The presence of amino groups in PCs fibrins accounts for this result [61]. In agreement with the results obtained, PRF exhibited the highest fibrin content compared to the other platelet concentrates. This characteristic likely contributed to its swelling rate, which did not compromise the structural integrity of the construct.

Regarding construct degradation after 48 hours, 10% PPP constructs exhibited the highest degradation rate (~80%), which was considered the highest average, and also 20% PRP resulted in 44% degradation. Likewise, Wang et al. studied the biological and mechanical characteristics of a PRP scaffold treated with 5%, 10%, and 15% calcium chloride (CaCl_2_). It was noted that 5% CaCl_2_ constructs lose approximately 37% of their mass on the third day. In contrast, the loss decreased to 30% and 12% with higher CaCl_2_ concentrations, respectively, indicating that a higher CaCl_2_ concentration used for crosslinking results in less mass loss [62]. Therefore, in our study, CaCl_2_ at 2.5% must have accounted for PRP degradation. On the other hand, Liu et al. described the degradation of cross-linked PRF at different genipin concentrations, a natural cross-linker with high biocompatibility (0.01, 0.1, 0.5, and 1%). The lowest degradation rate was observed for PRF without genipin, with a 55% mass loss at 24 h [63], suggesting that fibrinolysis occurs at a later time point and is responsible for PRF degradation. Furthermore, studies such as those performed by Barceló et al. highlight that supplementing other materials when printing alginate bioinks can reduce their degradation. Alginate, by itself, can degrade up to 50% after 48 hours, whereas adding 50% gelatin reduces degradation to 40% [64]. Hence, based on results reported in the literature, supplementation with PCs can diminish alginate construct degradation. However, degradation behavior varied depending on the type of platelet concentrate. While PRF-containing constructs exhibited more controlled degradation profiles, PPP supplementation accelerated mass loss, highlighting the influence of fibrin architecture on scaffold stability.

Lastly, regarding characterization by SEM, the 10% and 20% constructs and the 10% control with FBS all presented similar morphologies with rough surfaces. In contrast, 10% PRF revealed a more diverse morphology with wave-pattern filaments, which could have been acquired during the lyophilization. Such results agree with Irmark and collaborators’ study utilizing a 15% methacrylated gelatin (GelMA) with 50% PRP, observing an increased roughness with the presence of granules on the material’s surface and fibers corresponding to PC’s fibrin [65]. In the study by Ngah et al. (2021), similar structures were observed in lyophilized PRF, grouped into clusters. Nevertheless, clusters were not present in our study; we suppose they could have homogeneously integrated with the alginate, distributing the platelet component within the entire structure [66]. It is important to note that the SEM micrographs obtained in this study correspond to lyophilized constructs that were dehydrated under high-vacuum conditions before imaging. This process inevitably alters the hydrogel’s native morphology by collapsing the hydrated polymeric network and introducing surface artifacts. Therefore, the structures observed in SEM should be interpreted only as representations of the dry topology and not the true architecture of the hydrated constructs. To address this limitation, future work will incorporate cryogenic scanning electron microscopy (cryo-SEM), which allows imaging of frozen hydrated samples while preserving the native 3D microstructure and enabling more accurate characterization of pore distribution and fibrin network organization [67, 68].

In summary, this study provides several standalone findings. First, low supplementation levels of platelet concentrates, particularly 10% PRF, preserved pseudoplastic behavior while increasing viscosity, yield stress, and thixotropic recovery to values compatible with stable extrusion. Second, these rheological properties translated into scaffolds with good shape fidelity, controlled pore size, and manageable swelling and degradation profiles. Third, the incorporation of PCs, and especially PRF, significantly enhanced SaOS-2 viability after bioprinting compared with FBS, with 10% PRF constructs showing a continuous increase in viability up to 72 h. Together, these results support the use of platelet-enriched alginate bioinks, most notably the 10% PRF formulation, as a promising strategy for bone-related bioprinting applications.

This study also presents broader limitations that should be considered. The platelet concentrates were obtained from only two healthy donors, which limits the assessment of inter-donor variability. In addition, some analyses, such as rheological characterization, were performed in duplicate due to the limited availability of biological material. Although results were consistent, increasing the number of donors and replicates will improve statistical robustness and biological representativeness. Cell viability in 3D constructs was evaluated using Live/Dead imaging and MTS assays, which are widely used in bioprinting. Future studies could benefit from incorporating complementary viability and cytotoxicity assays, such as ATP quantification, total DNA content analysis, or luciferase-based metabolic activity tests, to provide additional confirmation of cellular responses in 3D environments. Finally, future *in vitro* and *in vivo* studies are required to quantify growth factor release, validate the osteogenic potential of the PRF-based bioinks, and further support their translational relevance for bone tissue regeneration.

## Conclusion

The 10% PRF and 10% PPP constructs demonstrated good performance in terms of swelling and degradation. Additionally, 10% PRF yielded the best cell viability after bio-printing with SaOS-2 cells, which was evaluated up to 72 hours; this outcome may be attributed to the controlled release of growth factors present in PCs. These characteristics, in addition to presenting ideal physical properties in terms of printability and rheology, suggest their greater suitability for bioprinting applications. Furthermore, the optimized PRF formulation allowed extrusion under low shear stress while preserving cell viability above 80%, underscoring the direct link between mechanical stress and cellular survival during and after printing. However, further studies, both *in vitro* and *in vivo*, are required to evaluate their performance in bone tissue regeneration. This study presents an innovative integration of platelet concentrates into bioinks, enhancing cell viability and material properties, which could transform bone tissue engineering and open new avenues for more effective regenerative therapies in clinical practice.

## Supplementary Information

Below is the link to the electronic supplementary material.Supplementary file1 (DOCX 10519 kb)

## Data Availability

The data supporting the findings of this study are available from the corresponding author upon reasonable request.
