# Supplementary material for: Bio-inks with PRF Increase Human Osteosarcoma Cell Line (SaOS-2) Viability in Extrusion-Based 3D-Bioprinted Constructs
Source: Ann Biomed Eng. 2026 Feb 25;54(9):2921–36. doi: 10.1007/s10439-026-04023-x (PMC13522032; doi:10.1007/s10439-026-04023-x)
Supplement: Supplementary file 1 [file 10439_2026_4023_MOESM1_ESM.docx]

**Supplementary Figure 1. Filament fusion test and printability evaluation of bioinks with platelet concentrates. (A)** Schematic representation of the filament fusion test used to evaluate print fidelity. Representative images of fused filaments printed with **(B)** 20% PRP, **(C)** 10% PPP, **(D)** 10% PRF, **(E)** 10% iPRF, **(F)** 20% iPRF**,** and **(G)** 10% FBS control**.** The measured fused filament thickness (ft) is indicated for each condition. **(H)** Diagram of the filament collapse platform with pillar distances ranging from 2 to 16 mm. Representative macroscopic images show the collapse behavior for **(I)** 20% PRP**, (J)** 10% PPP**, (K)** 10% PRF**, (L)** 10% iPRF**, (M)** 20% iPRF**,** and **(N)** control**.** Quantitative analysis of fusion fidelity, expressed as the fs/ft ratio, where values close to 1 indicate ideal printability and filament definition. **(O)** Bioinks supplemented with PRP 20%, PPP 10% y PRF 10% maintained fs/ft values within the acceptable range (1 ± 0.5), whereas iPRF 10% and 20% showed significantly higher ratios (**p* < 0.05), reflecting lower filament integrity and excessive spreading. These printability limitations supported the exclusion of iPRF formulations from subsequent 3D bioprinting steps.

**Supplementary Figure 2. Confocal microscopy micrographs showing live (green) and dead (red) SaOS-2 cells in bio-printed constructs at:** **(A, D, G, J)** corresponds at 24 h, **(B, E, H, K)** at 48 h, and **(C, F, I, L)** 72 h. **(S2A–S2C)** Constructs supplemented with 20% platelet-rich plasma (PRP), **(S2D–S2F)** 10% platelet-poor plasma (PPP), **(S2G–S2I)** 10% platelet-rich fibrin (PRF), and **(S2J–S2L)** 10% fetal bovine serum (FBS) control are shown.
